# Plaque psoriasis on the tongue: case report^☆^

**DOI:** 10.1016/j.abd.2024.01.008

**Published:** 2024-11-08

**Authors:** Lucas Campos Garcia, Nicole Vieira Schwan, Jésus Faria Rosa Júnior, Andrea Machado Coelho Ramos

**Affiliations:** a Department of Dermatology, Hospital das Clínicas, Universidade Federal de Minas Gerais, Belo Horizonte, MG, Brazil; b Department of Pathology, Hospital das Clínicas, Universidade Federal de Minas Gerais, Belo Horizonte, MG, Brazil

Dear Editor,

Although psoriasis is a disease with considerable prevalence in the Brazilian population, isolated oral involvement is rare. The histopathology of oral psoriasis was initially described in 1903 by Oppenheim,^1^ and over the years a few more reports have improved the literature on this subject.^2^, ^3^, ^4^ The diversity of clinical presentations and the occasional isolated occurrence, without association with skin lesions, are factors that complicate the diagnosis.^2^, ^3^ The present report describes a case of tongue psoriasis without associated skin involvement.

A previously healthy 45-year-old woman, a dentist, complained of a whitish plaque on her tongue for three months, with progressive increase. She showed no response to the use of triamcinolone acetonide orabase ointment for short periods or to the use of nystatin. She had a geographic tongue before the plaque appeared. She was an alcoholic and denied any family or previous history of psoriasis, continuous use of medication, or smoking. Physical examination revealed whitish plaques on the sides of the tongue (Fig. 1); there was no evidence of skin or skin appendage lesions. Histopathology (Fig. 2) showed hyperplastic squamous mucosal epithelium with elongation of the epithelial ridges, parakeratosis, and exocytosis of neutrophils with formation of Munro’s microabscesses. The lamina propria exhibited a predominantly lymphocytic inflammatory infiltrate, congested vessels, and edema. No fungi were identified and therefore the diagnosis was consistent with tongue psoriasis. Treatment involved interruption of alcohol consumption and betamethasone elixir three times a day and there was good progress after six months of treatment (Fig. 3).

**Figure 1:**
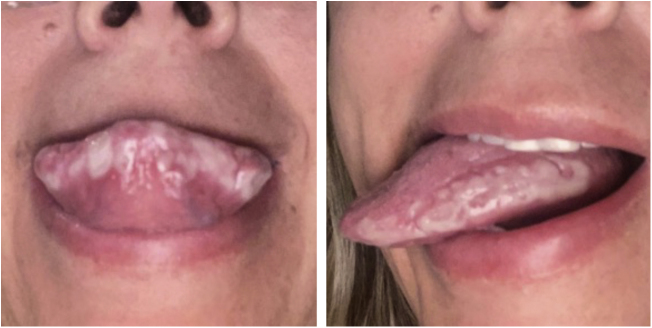
Whitish plaques on the tongue.

**Figure 2:**
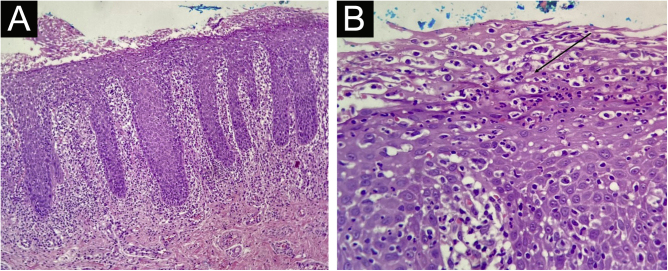
Histopathological findings of the tongue lesion. (A) Squamous mucosa showing hyperplastic psoriasiform epithelium (Hematoxylin & eosin, ×100). (B) Munro’s microabscess (arrow; Hematoxylin & eosin, ×400).

**Figure 3:**
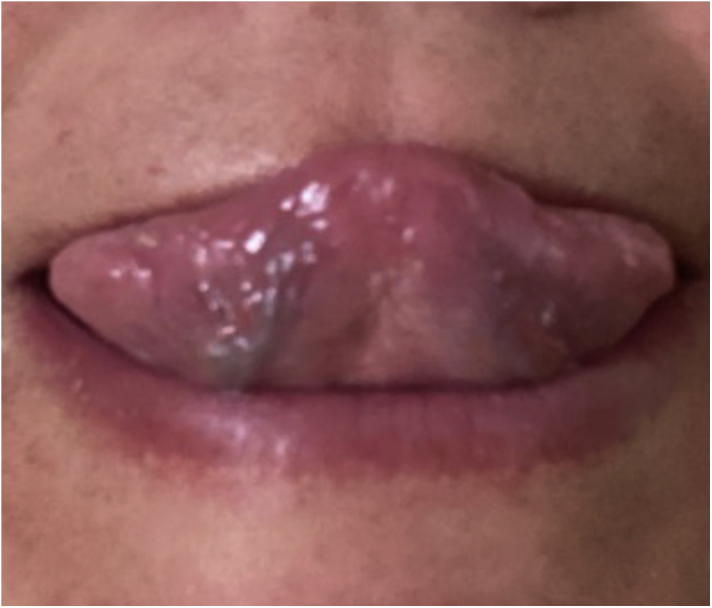
Total improvement of the plaques after alcohol consumption cessation treatment with local corticosteroids.

Psoriasis is a multifactorial and chronic disease, the etiology of which has yet to be completely elucidated. The most common oral mucosa findings are fissured tongue and geographic tongue, occasionally as an isolated manifestation.^2^, ^3^, ^4^ Other possible clinical presentations are yellowish-white or circinate plaques with histopathology compatible with psoriasis.^2^, ^3^ Oral psoriasis is frequently mistaken for other more common diseases, such as lichen planus, candidiasis, and syphilis, which makes the diagnosis challenging.^2^ In cases of isolated oral lesions, clinical suspicion must always be confirmed by anatomopathological examination.

Histopathology is similar to that of cutaneous psoriasis, with findings secondary to the hyperproliferation of keratinocytes: hyperkeratosis, parakeratosis and hypogranulosis, in addition to acanthosis with elongation of epidermal ridges. Two typical findings, resulting from neutrophil exocytosis, are the sterile accumulation in the stratum corneum (Munro's microabscess) and in the spinous layer (Kogoj's spongiform pustule). The superficial portion of the dermis most commonly exhibits lymphohistiocytic inflammatory infiltration and congested and tortuous blood vessels.^5^

Possible therapies include topical anesthetics, such as lidocaine or topical corticosteroids, as well as behavioral measures such as avoiding irritants such as alcohol, spicy foods, abrasion from dentures, and smoking.^2^ Eventually, systemic treatment may be necessary.

The present report describes a case of oral psoriasis in a patient without skin lesions but previously presenting geographic tongue. Histopathology was necessary to confirm the diagnosis and the patient showed good response to topical therapy. Isolated oral psoriasis is rare and possibly underdiagnosed. It is important that dermatologists be aware of this entity, especially as a differential diagnosis for other more prevalent diseases. The patient is under continuous follow-up every six months.

## Financial support

None declared.

## Authors' contributions

Lucas Campos Garcia: Statistical analysis; approval of the final version of the manuscript; design and planning of the study; drafting and editing of the manuscript; collection, analysis and interpretation of data; effective participation in research orientation; intellectual participation in the propaedeutic and/or therapeutic conduct of the studied cases; critical review of the literature; critical review of the manuscript.

Nicole Vieira Schwan: Statistical analysis; approval of the final version of the manuscript; design and planning of the study; drafting and editing of the manuscript; collection, analysis and interpretation of data; intellectual participation in the propaedeutic and/or therapeutic conduct of the studied cases; critical review of the literature; critical review of the manuscript.

Jésus Faria Rosa Júnior: Statistical analysis; approval of the final version of the manuscript; design and planning of the study; drafting and editing of the manuscript; collection, analysis and interpretation of data; critical review of the literature; critical review of the manuscript.

Andrea Machado Coelho Ramos: Statistical analysis; approval of the final version of the manuscript; effective participation in research orientation; intellectual participation in the propaedeutic and/or therapeutic conduct of the studied cases; critical review of the literature; critical review of the manuscript.

## Conflicts of interest

None declared.
